# BCRP expression does not result in resistance to STX140 *in vivo*, despite the increased expression of BCRP in A2780 cells *in vitro* after long-term STX140 exposure

**DOI:** 10.1038/sj.bjc.6604873

**Published:** 2009-01-20

**Authors:** J M Day, P A Foster, H J Tutill, S P Newman, Y T Ho, M P Leese, B V L Potter, M J Reed, A Purohit

**Affiliations:** 1 Department of Endocrinology and Metabolic Medicine and Sterix Ltd., Imperial College London, St Mary's Hospital, London W2 1NY, UK; 2 Department of Pharmacy and Pharmacology and Sterix Ltd., University of Bath, Bath BA2 7AY, UK

**Keywords:** ovarian cancer, 2-methoxyoestradiol, sulphamate, multidrug resistance, breast cancer resistance protein

## Abstract

The anti-proliferative and anti-angiogenic properties of the endogenous oestrogen metabolite, 2-methoxyoestradiol (2-MeOE2), are enhanced in a series of sulphamoylated derivatives of 2-MeOE2. To investigate possible mechanisms of resistance to these compounds, a cell line, A2780.140, eightfold less sensitive to the 3,17-*O*,*O*-bis-sulphamoylated derivative, STX140, was derived from the A2780 ovarian cancer cell line by dose escalation. Other cell lines tested did not develop STX140 resistance. RT–PCR and immunoblot analysis demonstrated that breast cancer resistance protein (BCRP) expression is dramatically increased in A2780.140 cells. The cells are cross-resistant to the most structurally similar bis-sulphamates, and to BCRP substrates, mitoxantrone and doxorubicin; but they remain sensitive to taxol, an MDR1 substrate, and to all other sulphamates tested. Sensitivity can be restored using a BCRP inhibitor, and this pattern of resistance is also seen in a BCRP-expressing MCF-7-derived cell line, MCF-7.MR. In mice bearing wild-type (wt) and BCRP-expressing tumours on either flank, both STX140 and mitoxantrone inhibited the growth of the MCF-7wt xenografts, but only STX140 inhibited growth of the MCF-7.MR tumours. In conclusion, STX140, a promising orally bioavailable anti-cancer agent in pre-clinical development, is highly efficacious in BCRP-expressing xenografts. This is despite an increase in BCRP expression in A2780 cells *in vitro* after chronic dosing with STX140.

The human endogenous metabolite, 2-methoxyoestradiol (2-MeOE2), has been shown to inhibit the proliferation of many cancer cell types *in vitro* (Zhu and Conney, 1998) and *in vivo* (Klauber *et al*, 1997), and to inhibit angiogenesis (Fotsis *et al*, 1994). 2-MeOE2 binds to the colchicine binding site of tubulin (D’Amato *et al*, 1994) inhibiting tubulin polymerisation. This results in the arrest of the cells in the G_2_/M phase of the cell cycle and leads to subsequent apoptosis (Qadan *et al*, 2001).

The anti-proliferative properties of 2-MeOE2 are enhanced in a series of 2-substituted sulphamoylated oestradiol derivatives (Raobaikady *et al*, 2003; Ireson *et al*, 2004; Newman *et al*, 2004; Leese *et al*, 2005a, 2005b). As with 2-MeOE2, the sulphamoylated compounds interact with tubulin, leading to G_2_/M phase cell cycle arrest and apoptosis (MacCarthy-Morrogh *et al*, 2000). One of the most efficacious of these compounds is 2-methoxyoestradiol-3,17-*O,O*-bis-sulphamate (STX140). It is highly anti-proliferative and anti-angiogenic *in vitro* (Raobaikady *et al*, 2003; Newman *et al*, 2004), and is orally bioavailable (Ireson *et al*, 2004; Newman *et al*, 2006), potently inhibiting tumour growth (Utsumi *et al*, 2005; Foster *et al*, 2008) and angiogenesis (Chander *et al*, 2007) *in vivo*. STX140 is currently in pre-clinical development as a novel anti-cancer therapy (Newman *et al*, 2007).

The development of drug resistance is a major hurdle in the treatment of cancer. Although many tumours initially respond to therapy, a large number become resistant over time. Mechanisms of resistance range from changes in accumulation of the drug within the cell, and mutations in enzymes and targets, to induction of cellular compensation mechanisms. These changes can result in the cells becoming resistant to more than one drug, a phenomenon known as multi-drug resistance. A common mechanism of multi-drug resistance is the enhanced expression of various ATP-binding cassette (ABC) membrane transporter proteins, including the well-studied P(170)-glycoprotein/multidrug resistance protein 1/MDR1 (Juliano and Ling, 1976; Ambudkar *et al*, 2003), the multi-drug resistance-related protein/MRP/MOAT family (Kruh and Belinsky, 2003), and breast cancer resistance protein/BCRP/ABCG2/MXR (Doyle and Ross, 2003). These membrane-based proteins have different substrate specificities, but all decrease drug accumulation within cells by rapid energy-dependent drug efflux.

Previous work from our group has established that STX140 is efficacious in P-glycoprotein-expressing tumours derived from MCF-7 breast cancer cells, MCF-7_DOX_, both *in vitro* (Suzuki *et al*, 2003) and *in vivo* (Newman *et al*, 2008). We have also looked at the effects of STX140 and other sulphamoylated 2-MeOE2 derivatives on two drug-resistant sub-lines derived from the ovarian cell line, A2780. These include an adriamycin (DOX)-resistant sub-line, A2780adr (Hamilton *et al*, 1984), which also over-expresses P-glycoprotein (Van der Bliek *et al*, 1988), and a cisplatin-resistant A2780-derived cell line, A2780cis (Behrens *et al*, 1987), whose suggested mechanisms of resistance include loss of the DNA mismatch repair pathway (Aird *et al*, 2002), changes in glutathione content (Godwin *et al*, 1992), and intracellular sequestration of cisplatin (Kalayda *et al*, 2004), amongst others (Siddik, 2003). The potency of the sulphamates is unchanged in the cisplatin-resistant sub-line in comparison to the A2780wt cell line. The efficacy is only 2–3 times lower in the P-glycoprotein-expressing A2780adr cell line than in the parental line (Day *et al*, 2003), and this is similar to the 1.5-fold change in efficacy seen between the P-glycoprotein-expressing MCF-7_DOX_ cells and the MCF-7wt cells (Newman *et al*, 2008). These values compare very favourably to the effects of P-glycoprotein on agents such as Taxol and DOX, whose efficacies are >150-fold lower in P-glycoprotein-expressing MCF-7 cells (Newman *et al*, 2008). STX140 is also efficacious in xenografts derived from patient tumours which are drug-resistant but which do not express P-glycoprotein (Newman *et al*, 2008). This suggests that the sulphamoylated compounds overcome many cellular multi-drug resistance mechanisms and may be effective against tumours, which are already resistant to conventional chemotherapeutic regimens.

In this study we describe the *in vitro* derivation and characterisation of an A2780-based cell line resistant to STX140, designated A2780.140. We demonstrate that although the resistance of the A2780.140 cells appears to be due to the increased expression of BCRP, this only causes an eightfold decrease in the potency of STX140 *in vitro*, and does not affect the efficacy of STX140 *in vivo*.

## Materials and methods

### Drug synthesis

**Figure 1:**
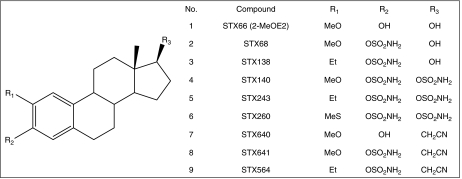
The structures of the 2-methoxyoestradiol derivatives. (1) 2-MeOE2 (STX66; 2-methoxyoestradiol); (2) STX68 (2-methoxyoestradiol-3-*O*-sulphamate); (3) STX138 (2-ethyloestradiol-3-*O*-sulphamate); (4) STX140 (2-methoxyoestradiol-3,17-*O*,*O*-bis-sulphamate); (5) STX243 (2-ethyloestradiol-3,17-*O*,*O*-bis-sulphamate); (6) STX260 (2-methylsulphanyloestradiol-3,17-*O*,*O*-bis-sulphamate); (7) STX640 (2-methoxy-3-hydroxy-17*β*-cyanomethyl-estra-1,3,5(10)-triene); (8) STX641 (2-methoxy-3-*O*-sulphamoyl-17*β*-cyanomethyl-estra-1,3,5(10)-triene); (9) STX564 (2-ethyl-3-*O*-sulphamoyl-17*β*-cyanomethyl-estra-1,3,5(10)-triene).

**Figure 2:**
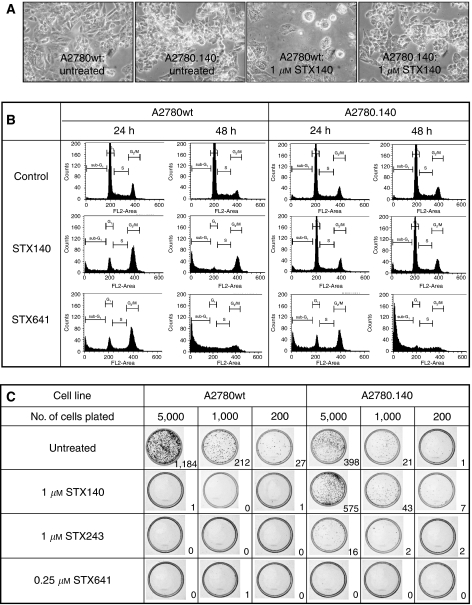
A2780wt and A2780.140 cells after treatment with 1 *μ*M sulphamoylated oestradiol derivatives. (**A**). Light microscopy of A2780wt and A2780.140 cells, either untreated or treated with 1 *μ*M STX140 for 72 h (40 × ). (**B**) Cell cycle profiles of A2780wt and A2780.140 cells treated with 1 *μ*M STX140 or STX641 for 24 or 48 h: cells harvested by trypsinisation were pelleted, washed twice with PBS, fixed in cold 70% ethanol, treated with 100 *μ*g ml^−1^ RNase for 5 min, stained with 50 *μ*g ml^−1^ propidium iodide and analysed using a flow cytometer (FACScan; Becton Dickinson). (**C**) The effect of STX140, STX243 and STX641 on the clonogenic potential of A2780wt and A2780.140 cells: cells at low confluency were left untreated as a control, or treated with 1 *μ*M STX140, 1 *μ*M STX243 or 0.25 *μ*M STX641. After 72 h, cells were washed thoroughly, replated at 5000, 1000 or 200 cells per 60 mm^2^ dish and allowed to grow until colonies became established. The colonies were then Giemsa stained, photographed and counted (no. of colonies are shown in bottom right hand corner of each panel).

**Figure 3:**
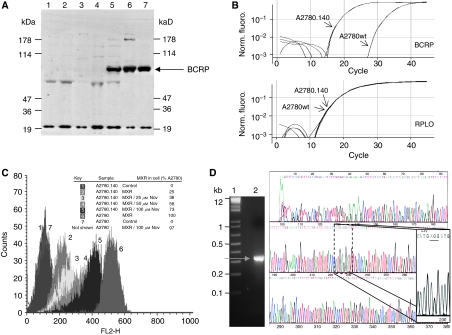
Analysis of BCRP expression and functionality in A2780.140 cells. (**A**) Expression of BCRP in A2780 cells during the development of resistance to STX140: cells were harvested and solubilised using RIPA buffer. Cell extracts (15 *μ*g) separated by electrophoresis on 4–12% NuPAGE gels (Invitrogen) and transferred to nitrocellulose were immunoblotted with anti-BCRP mAb (MAB4146; CHEMICON International Inc.) at 1 : 200. Lane 1: A2780wt cells treated with THF (vehicle) for 24 h; lane 2: A2780wt cells treated with 1 *μ*M STX140 for 24 h; lanes 3–7: A2780.140 cells treated with STX140; lane 3: 100 nM (early dose); lane 4: 100 nM (constant dose); lane 5: 500 nM (early dose); lane 6: 500 nM (constant dose); lane 7: 1 *μ*M (constant dose). Markers=MultiMark (Invitrogen). (**B**) Expression of BCRP mRNA in A2780wt and A2780.140 cells: RT–PCR analysis of mRNA from untreated A2780wt and A2780.140 cells using Taqman expression assays (Applied Biosystems) containing primers and probes for BCRP, and for an endogenous control gene, RPLO. (**C**) The effect of Nov on the accumulation of mitoxantrone (MXR) in A2780wt and A2780.140 cells: cells pre-treated with Nov were treated with 10 *μ*M MXR +/− Nov, harvested by trypsinisation and resuspended in ice-cold PBS with 2.5% fetal calf serum. MXR accumulation was analysed using a flow cytometer (FACScan; Becton Dickinson) with excitation and emission wavelengths of 633 and 661 nm, respectively, and the relative amount of MXR in each sample was calculated as a percentage of the median linear fluorescence in the A2780wt + MXR samples (A2780=A2780wt; representative of two separate experiments). (**D**) The wild-type form of BCRP, R482, is expressed by the A2780.140 cells. A 316 bp fragment of BCRP was amplified by RT–PCR (grey arrow), purified and sequenced. Lane 1: 1 kb plus marker (Invitrogen); lane 2: A2780.140 PCR product: the sequence of this product is shown to the right hand side of the gel.

**Figure 4:**
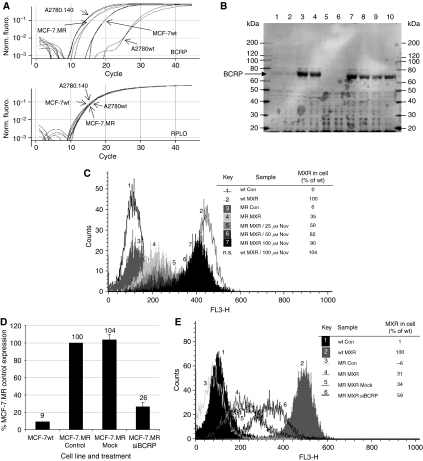
Analysis of BCRP expression and functionality in MCF-7.MR cells. (**A**) Expression of BCRP mRNA in MCF-7wt and A2780wt cells, and in the derivatives, MCF-7.MR and A2780.140: RT–PCR analysis of mRNA from untreated cells using Taqman expression assays (Applied Biosystems) containing primers and probes for BCRP, and for an endogenous control gene, RPLO. (**B**) Expression of BCRP protein in MCF-7wt and A2780wt cells, and in the derivatives, MCF-7.MR and A2780.140: protein from untreated cells was analysed by immunoblot as in Figure 3A. Lanes 1 and 2: MCF-7wt; lanes 3 and 4: MCF-7.MR; lanes 5 and 6: A2780wt; lanes 7 and 8: A2780.140; lanes 9 and 10: A2780.140 (no STX140 treatment for 8 weeks). Markers=MagicMark (Invitrogen). (**C**) The effect of Nov on the accumulation of MXR in MCF-7wt and MCF-7.MR cells: cells pre-treated with Nov were treated with 10 *μ*M MXR +/− Nov, harvested by trypsinisation and resuspended in ice-cold PBS with 2.5% fetal calf serum. MXR accumulation was analysed using a flow cytometer (FACScan; Becton Dickinson) as in Figure 3C (wt=MCF-7wt; MR=MCF-7.MR; representative of two separate experiments). (**D**) Expression of BCRP mRNA in MCF-7.MR cells after BCRP siRNA transfection: RT–PCR analysis of mRNA from MCF-7wt and MCF-7.MR cells 48 h after transfection with 3 *μ*g BCRP siRNA (Ambion, UK) using Taqman expression assays for BCRP, and for an endogenous control gene, RPLO. (**E**) The effect of BCRP siRNA transfection on the accumulation of MXR in MCF-7.MR cells: 48 h after transfection with 3 *μ*g BCRP siRNA (Ambion, UK), MCF-7wt and MCF-7.MR cells were treated with 10 *μ*M MXR +/− Nov, harvested by trypsinisation and resuspended in ice-cold PBS with 2.5% fetal calf serum. MXR accumulation was analysed using a flow cytometer (FACScan; Becton Dickinson) as in Figure 3C (wt=MCF-7wt; MR=MCF-7.MR; representative of two separate experiments).

**Figure 5:**
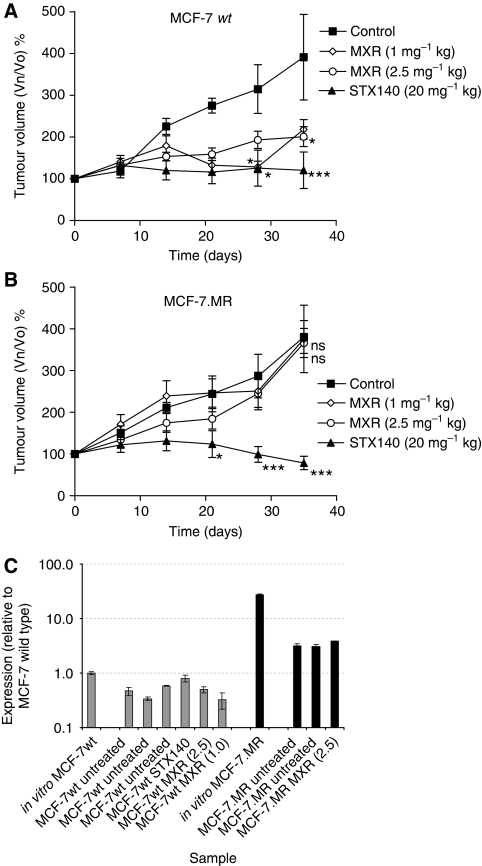
Dual tumour MCF-7wt and MCF-7.MR xenograft model. Female MF-1 nu/nu mice were injected s.c. in one flank with 5 × 10^6^ MCF-7wt cells and in the other with 5 × 10^6^ MCF-7.MR cells in ice-cold Matrigel (*n*=6 per group). Daily oral administration of STX140 vehicle (0.1 ml 10% THF/90% propylene glycol), STX140 (20 mg kg^−1^), or twice weekly i.v. administration of MXR (1.0 and 2.5 mg kg^−1^ in saline) was initiated when the tumours reached 50–150 mm^3^ in volume (day 0). (**A**) MCF-7wt tumour growth: dosing with STX140 or either dose of MXR caused significant inhibition of tumour growth (^***^P<0.001 and ^*^P<0.05, respectively) compared to control. (**B**) MCF-7.MR tumour growth: dosing with STX140 caused significant inhibition of tumour growth (^***^P<0.001) compared to control, whereas dosing with MXR at either dose did not affect tumour growth (ns, *P*>0.05). (**C**) BCRP mRNA expression: RT–PCR analysis, using Taqman expression assays for BCRP, and for an endogenous control gene, RPLO, of mRNA extracted from MCF-7wt and MCF-7.MR tumours at the end of the study.

The syntheses of the 2-MeOE2 derivatives have been reported previously: 2-MeOE2 (STX66; Figure 1, **1**), 2-methoxyoestradiol-3-*O*-sulphamate (STX68; Figure 1, **2**) and 2-ethyloestradiol-3-*O*-sulphamate (STX138; Figure 1, **3**), by Leese *et al* (2005b); the bis-sulphamoylated oestradiol derivatives, 2-methoxyoestradiol-3,17-*O,O*-bis-sulphamate (STX140; Figure 1, **4**), 2-ethyloestradiol-3,17-*O,O*-bis-sulphamate (STX243; Figure 1, **5**) and 2-methylsulphanyloestradiol-3,17-*O,O*-bis-sulphamate (STX260; Figure 1, **6**), by Leese *et al* (2006); and the 2-methoxy- and 2-ethyl-3-hydroxy-17*β*-cyanomethyl-estra-1,3,5(10)-triene derivatives, STX640 (Figure 1, **7**), STX641 (Figure 1, **8**) and STX564 (Figure 1, **9**), by Leese *et al* (2008).

### Cell culture

Cell culture medium and supplements were purchased from the Sigma-Aldrich Company Ltd. (Dorset, UK). The A2780 (A2780wt) ovarian carcinoma cancer cell line was purchased from the European Collection of Cell Cultures (ECACC, Wiltshire, UK), and the MCF-7 (MCF-7wt) breast cancer cell line from the American Tissue Culture Collection (ATCC, Middlesex, UK), and both were grown in RPMI supplemented with 10% FBS, 2 mM L-glutamine, 1% non-essential amino acids and 0.075% sodium bicarbonate. The cells were maintained at 37°C in a humidified atmosphere at 5% CO_2_. The MCF-7.MR cell line, an MCF-7 derivative resistant to mitoxantrone (MXR), was a kind gift from Dr GL Scheffer (Department of Pathology, Free University Hospital, Amsterdam, the Netherlands). To maintain the resistance of the MCF-7.MR cells, they were cultured in 80 nM MXR (MXR dihydrochloride in PBS; M6545; Sigma).

### Derivation of an STX140-resistant sub-line of ovarian A2780 cells

The STX140-resistant sub-line, A2780.140, was derived by exposure of the A2780wt cell line to increasing concentrations of STX140 over 3 months, with non-treatment recovery periods, from 100 nM to a final constant concentration of 1 *μ*M. Stock A2780.140 cells were then maintained in 1 *μ*M STX140. At least 72 h before each experiment STX140 was removed from the medium, and the cells were washed and cultured in fresh untreated medium.

### Proliferation assay

Logarithmically growing cells were plated onto 96-well plates (Falcon, Marathon Lab Supplies, London, UK) at a density of 5–6 × 10^3^ cells per well four hours before treatment with a range of concentrations of test compound in tetrahydrofuran (THF; Sigma) vehicle. Control cells received 0.2% THF, a dose equivalent to 10 *μ*M compound. After incubation for 96 h the CellTiter96 Aqueous One assay reagent (Promega, Hampshire, UK) was added to measure cell proliferation. The cells were incubated for a further 2–4 h at 37°C before the absorbance of the wells was measured at 492 nm. The IC_50_ was calculated for each compound using Prism software (version 3.02). The relative sensitivity of the resistant cell line to each compound compared to that of the parent cell line was calculated as the resistance factor (R.F.=IC_50_ resistant cell line/IC_50_ wt cell line).

### Cell morphology

#### Light microscopy

Unfixed cells were photographed 72 h after treatment with 1 *μ*M STX140. The photographs were taken on a Kodak DC120 digital camera with an Olympus CK2 microscope (Olympus UK Ltd., Middlesex, UK) and processed with Adobe Photoshop 5.0LE.

### Cell-cycle flow cytometric analysis

Cells were plated at 50–60% confluency in T25 flasks (Triple Red, Oxfordshire, UK). After 24 h they were treated with 1 *μ*M of STX140 or STX641. After a further 24 or 48 h the cells were harvested by trypsinisation. All media and washings were collected. The cells and washings were pelleted by centrifugation at 1100 *g*, washed twice with PBS, fixed in cold 70% ethanol, treated with 100 *μ*g ml^−1^ RNase for 5 min, stained with 50 *μ*g ml^−1^ propidium iodide and analysed using a flow cytometer (FACScan; Becton Dickinson, Oxfordshire, UK).

### Clonogenicity assay

Cells were plated in T25 flasks (Triple Red) at low confluency (∼40%). After 24 h they were treated with concentrations of STX140, STX243 or STX641 that were approximately four times the IC_50_ value obtained from the proliferation assay (1 *μ*M STX140 or STX243, or 0.25 *μ*M STX641). Control cells were untreated. After 3 days the cells were trypsinised and resuspended in 5 ml medium in the absence of treatments. The cell suspension was counted using a haemocytometer and the cells replated in 60mm^2^ gridded dishes at 5000, 1000, and 200 cells per dish (Corning; Fisher Scientific UK Ltd., Leicestershire, UK). These were cultured for 8 days. When colonies became visible and cells became confluent in higher density dishes, the colonies were fixed in cold methanol for 30 min, stained in 1 : 10 Giemsa stain in water for 10 min (Accustain: Giemsa stain, modified; Sigma; GS-500), rinsed twice in water, photographed (Kodak DC290) and the number of colonies counted (Kodak 1D version 3.5; Eastman Kodak Company, Stamford, CT, USA).

### Immunoblot analysis of cell lysates

A2780, A2780.140, MCF-7 and MCF-7.MR cells were treated with various doses of STX140 for varying durations. Medium was removed and treated cells were scraped from the flask, washed with PBS and lysed with RIPA buffer (250 mM Tris-HCl pH 8.0, 750 mM NaCl, 5% Nonidet P40, 2.5% sodium deoxycholate, 0.5% SDS) in the presence of protease and phosphatase inhibitors (1 mM PMSF, 1 mM EDTA, 5 *μ*g ml^−1^ aprotinin, 5 *μ*g ml^−1^ leupeptin). The non-soluble material was removed by centrifugation. Protein concentration was determined using the Bradford assay (Bio-Rad Laboratories, Hertfordshire, UK), and 15 *μ*g samples were separated by electrophoresis under reducing conditions on 4–12% Bis-Tris NuPAGE gels (Invitrogen, Paisley, UK) before being transferred to nitrocellulose membranes. Equal sample loading and transfer were confirmed by Ponceau R staining (Sigma). Filters were immunoblotted with the required monoclonal antibody in incubation buffer containing 0.1% milk (Marvel; Premier Brands UK Ltd., Lincolnshire, UK) in PBS. Bound antibody was detected with horseradish peroxidase-conjugated anti-mouse secondary antibody and chemiluminescence (SuperSignal West Dura substrate; Perbio Science UK Ltd., Northumberland, UK).

### Real-time RT-PCR

Total mRNA was purified from T75 flasks at approximately 80% confluency using QIAshredder and RNeasy kits (QIAGEN, West Sussex, UK) and stored at −80°C. A 5 *μ*g aliquot of each mRNA sample was reverse transcribed in a final volume of 33 *μ*l to generate cDNA using the ‘First-Strand cDNA Synthesis Kit’ (GE Healthcare Ltd., Buckinghamshire, UK) and stored at −20°C. RT–PCR reactions were performed in a ‘Rotor Gene 2000 Real-Time Cycler’ (Corbett Research, Cambridgeshire, UK) with 0.5 *μ*l cDNA in a final volume of 10 *μ*l, using Taqman universal PCR master mix and Taqman expression assays containing primers and probes for BCRP, and for an endogenous control gene, RPLO (Applied Biosystems, Warrington, UK). The conditions were as follows: 95°C for 10 min, followed by 40 cycles of denaturation at 95°C for 15 s and annealing/amplification at 60°C for 60 s in accordance with the recommended conditions for these primers and probes (Applied Biosystems). The expression of mRNA for other multi-drug resistance proteins was analysed using 1 *μ*l cDNA in a final volume of 25 *μ*l, in Excite 2 × Master Mix (Biogene, Cambridgeshire, UK) and primers for either MDR1, MRP1, MRP2, MRP3 (Kawabata *et al*, 2001), with the endogenous gene, GAPDH as an internal control (forward primer 5′-TGCCGTCTAGAAAAACCTGC-3′; reverse primer 5′-ACCCTGTTGCTGTAGCCAAA-3′). The RT–PCR conditions were as follows: 95°C for 10 min; followed by 40 cycles of denaturation at 95°C for 30 s, annealing at 55°C for 45 s, and amplification at 72°C for 45 s. Relative mRNA expression was calculated using the comparative quantitation algorithm in the Rotor Gene 6 software (Corbett Life Science, Corbett Research UK, Cambridgeshire, UK).

### Substrate accumulation flow cytometric analysis

Cells were plated at 100–150 × 10^3^ cells per well in 12-well plates (Triple Red). After 24 h they were pre-treated, if pre-treatment was required, with 0–100 *μ*M novobiocin (Nov: sodium salt in DMSO; N6160; Sigma) for 1 h before treatment with 10 *μ*M MXR in either the presence or absence of Nov. After a further hour the cells were harvested by trypsinisation, and placed immediately on ice. Collected cells were pelleted by centrifugation at 1100 *g*, resuspended in ice-cold PBS with 2.5% fetal calf serum, and the accumulation of MXR analysed using a flow cytometer (FACScan; Becton Dickinson) with excitation and emission wavelengths of 633 and 661 nm, respectively. The relative amount of MXR in each sample was calculated as a percentage of the median linear fluorescence in the wt + MXR samples using the wt control (no MXR) samples as a blank.

### BCRP amplification and sequencing

An aliquot of cDNA product from the A2780.140 cell line mRNA (see section ‘Real-time RT-PCR’) was used for PCR using BCRP primers (Kawabata *et al*, 2001). The 0.5 *μ*l cDNA aliquot was amplified using 35 cycles of: denaturation at 95°C for 30 s, annealing at 58.5°C for 45 s, and extension at 72°C for 1 min. The product was separated by electrophoresis on a 1.5% agarose gel containing ethidium bromide, and the band of 316 bp visualised under UV light, excised from the gel, purified (QIAquick gel extraction kit; QIAGEN) and sequenced (PE Biosystems, Buckinghamshire, UK).

### siRNA studies

MCF-7.MR cells (1 × 10^6^) were transfected with 3 *μ*g of BCRP siRNA (Ambion Inc., Austin, TX, USA) using the AMAXA system with Nucleofector kit V (AMAXA, Cologne, Germany) according to the manufacturer's protocol. The cells were maintained in a 6-well plate and 48 h post-transfection BCRP mRNA expression and accumulation of MXR by the cells was assayed.

### Xenograft models

Animal experiments were approved by the Imperial College Ethical Review Committee and were conducted in accordance with the UK Animals (Scientific Procedures) Act (1986) and the UKCCCR guidelines for the Welfare of Animals in Experimental Neoplasia (Workman *et al*, 1998). All efforts were made to minimise both suffering and the number of animals used.

Female MF-1 nu/nu mice (Harlan UK Ltd., Oxfordshire, UK) were injected s.c. in one flank with 5 × 10^6^ A2780wt or MCF-7wt cells and in the other with 5 × 10^6^ A2780.140 or MCF-7.MR cells respectively in ice-cold Matrigel (0.1 ml), resulting in a single tumour per flank. Daily oral administration of STX140 (20 mg kg^−1^) in 0.1 ml 10% THF/90% propylene glycol or twice weekly i.v. administration of 1 or 2.5 mg kg^−1^ MXR in 0.1 ml saline was initiated when the tumours reached 50–150 mm^3^ in volume (*n*=4–6 per group). Control animals were dosed orally with 10% THF/90% propylene glycol. Animal weights and tumour measurements were recorded every 7 days. Tumour volume (*V*), in mm^3^, was determined using the following equation: *V*=length × width^2^/2. Results are expressed as a percentage of the tumour volume at day of measurement (Vn) over the volume at day 0 (Vo). At the end of study, the animals were sacrificed and tumour tissue (20–40 mg) was excised and transferred to RNAlater solution (Ambion) for subsequent RNA purification.

### Statistical analysis

*In vitro* experiments were carried out in triplicate and data presented are representative of one of three such experiments. Errors shown are the mean±s.d. ANOVA was used to assess the significance in *in vivo* data. *In vivo* study data are represented as mean±s.e.m.

## Results

A2780.140, a cell line resistant to STX140 (Figure 1, **4**), was derived from A2780 ovarian cancer cells. After 3 months of increasing the dose of STX140 from 100 nM to 1 *μ*M, allowing time for recovery after each dose escalation, the cells could be cultured constantly in 1 *μ*M STX140. Parallel attempts to establish STX140-resistant sub-lines from other cell types, including MCF-7, PC-3 and LNCaP cells, all with a similar sensitivity to STX140 as the parental A2780 cells, were unsuccessful.

The sensitivity of the A2780wt and A2780.140 cells to various compounds, both sulphamates and other chemotherapeutic agents, was measured using a tetrazolium dye assay, and the IC_50_ values and resistance factors (R.F.) were calculated (Table 1). The A2780wt is eight times more sensitive to STX140 than the resistant line. The A2780.140 cell line is also cross-resistant to two structurally similar bis-sulphamoylated derivatives, STX260 (2-methylsulphanyloestradiol-3,17-*O,O*-bis-sulphamate; Figure 1, **6**) and STX243 (2-ethyloestradiol-3,17-*O,O*-bis-sulphamate; Figure 1, **5**) although with a lower resistance factor (R.F. of 5.0 and 3.2, respectively). These compounds differ from STX140 only in the substitution at their 2-positions. There is no difference in sensitivity to all other sulphamoylated compounds in the series between the two cell lines, or to colchicine or the P-glycoprotein substrate, taxol. However, the A2780.140 line is resistant to MXR, with an R.F. of 6.5, and to some extent to DOX, with an R.F. of 3.0.

The morphology of the A2780 and A2780.140 cells is shown in Figure 2A. After 72 h of treatment with 1 *μ*M STX140, the A2780wt cells became detached and rounded, displaying the characteristic appearance of cells undergoing apoptosis, whereas the morphology of the A2780.140 line was unaffected by the treatment. Flow cytometry was used to assess the effects of 1 *μ*M STX140 and another sulphamoylated derivative, STX641 (2-methoxy-3-*O*-sulphamoyl-17*β*-cyanomethyl-estra-1,3,5(10)-triene), on the cell cycle of the cell lines over 48 h (Figure 2B). In the A2780wt cells there was a marked increase in the G_2_/M peak after 24 h of treatment with either compound when compared to untreated cells, with a subsequent rise in the sub-G_1_ population over the following 24 h. These effects were also apparent in the A2780.140 cells after treatment with STX641, but not after treatment with 1 *μ*M STX140: in A2780.140 cells treated with STX140 there was no change in cell-cycle distribution when compared to untreated A2780.140 cells.

To confirm that the A2780.140 cells are resistant to the long-term growth inhibitory effects of STX140, we studied the effect of STX140, STX243 and STX641 on the clonogenic potential of A2780.140 and A2780wt cells. Figure 2C shows the Giemsa-stained colonies formed by cells which were replated and allowed to grow in untreated complete medium after prior 72 h treatment with the compounds at concentrations of approximately four times their IC_50_. The wild-type cell line was very susceptible to the long-term effects of these compounds, with all treatments resulting in no more than 1 colony per plate, although the untreated control plates, at plating dilutions of 5000, 1000 and 200 cells, contained 1184, 212 and 27 colonies, respectively. However, in the resistant cell line, the cells treated with STX140 grew at least as well as the untreated cells, indicating that these cells are resistant to the long-term effects of STX140 at 1 *μ*M. There was also some resistance to STX243, but not to STX641, with no colonies forming even after treatment at 0.25 *μ*M, in agreement with the results of the proliferation assays.

Anti-BCRP mAb (MAB4146 at 1 : 200; CHEMICON International, Inc., Hampshire, UK) immunoblot analysis of cell lysates prepared from A2780 cells during derivation of the A2780.140 cell line indicated that there is significant expression of BCRP in all cells resistant to ⩾500 nM STX140 (Figure 3A), but that there is no BCRP band visible in the A2780wt lanes. No change in expression of other multi-drug resistance proteins, including P-glycoprotein (MDR1), multi-drug resistance-associated protein (MRP1) and lung resistance protein (LRP) could be detected by immunoblotting (results not shown). Real-time RT-PCR of mRNA from A2780wt and A2780.140 cell lines confirmed that BCRP mRNA expression is negligible in A2780wt cells, but is upregulated approximately 700-fold (calculated from triplicate RT–PCR experiments) in the A2780.140 cells (Figure 3B). There was no change in mRNA expression of other multi-drug resistance proteins including MDR1, MRP1, MRP2 and MRP3 (results not shown).

The effect of 60 *μ*M Nov, a specific inhibitor of BCRP (Shiozawa *et al*, 2004), on the sensitivities of the two cell lines to various compounds is shown in the last column of Table 1. In the presence of Nov the sensitivity of the A2780.140 cell line to STX140, STX260, MXR and DOX was restored almost to that of the A2780wt cell line whereas the sensitivity of A2780.140 cells to STX641 was unaffected. This suggests that BCRP is the only major mechanism of resistance in the resistant cell line. To confirm the activity of BCRP in these cells, FACS analysis was used to study the effect of Nov on the accumulation of MXR, a fluorescent BCRP substrate, in the A2780.140 cells (Figure 3C). The fluorescence of the cells is low after incubation with MXR alone; however, in the presence of increasing concentrations of Nov, the fluorescence of the cells increases from 25 to 73% of the level in the MXR-incubated A2780wt cells, due to the inhibition of BCRP-mediated MXR efflux.

The substrate specificity of BCRP is dependent on the amino acid which is present at position 482; in the wild-type this is an arginine, but BCRP can also be expressed with either a threonine or a glycine residue at this position (Lee *et al*, 1997; Miyake *et al*, 1999). To establish which form of BCRP is being over-expressed by the A2780.140 cell line, a 316 bp fragment of BCRP was amplified by RT–PCR, purified and sequenced (Figure 3D). This sequence established that the wild-type form of BCRP, R482, is expressed by the A2780.140 cells.

As further confirmation that BCRP expression is the major method of resistance in the derived cell line, the A2780.140 cell line was compared to a known BCRP over-expressing cell line, MCF-7.MR. RT–PCR analysis (Figure 4A) indicated that, as expected, BCRP mRNA is highly expressed in the MCF-7.MR cells, at an approximately 10-fold higher level than in MCF-7wt cells, and at a similar level to the A2780.140 cells. The A2780wt cells have the lowest BCRP mRNA expression of all four cell lines. Immunoblot analysis of BCRP protein expression across the four cell lines (Figure 4B) confirmed that BCRP expression in the A2780wt cells is negligible (lanes 5 and 6), with some expression apparent in the MCF-7wt cell line (lanes 1 and 2), whereas both A2780.140 (lanes 7 and 8) and MCF-7.MR (lanes 3 and 4) express a similarly high level of BCRP. The high level of BCRP expression is maintained in A2780.140 cells cultured for eight weeks in the absence of STX140 (lanes 9 and 10). This was confirmed by RT–PCR of mRNA samples taken from these cells (results not shown).

Substrate accumulation flow cytometric analysis of the effect of Nov on the accumulation of MXR in MCF-7.MR cells (Figure 4C) indicated that the established functionality and inhibition of BCRP in this line is very similar to that in A2780.140 cells, with the presence of 100 *μ*M Nov resulting in 90% of the MXR accumulation of the wild-type cells. Transfection of the MCF-7.MR cells with BCRP siRNA caused a 4-fold decrease in the expression of BCRP mRNA (Figure 4D), which was reflected in an increase in accumulation of MXR, from 31 to 59% of that in MCF-7wt cells when assayed using the substrate accumulation flow cytometric assay (Figure 4E). The sensitivity of the MCF-7.MR and MCF-7wt cell lines to several of the sulphamoylated compounds, and to MXR, was determined (Table 2). The pattern of resistance to the compounds is the same as in the A2780.140 cell line, with the MCF-7.MR cell line having increased resistance to STX140 (R.F. 6.4), MXR (R.F. 5.4), STX243 (R.F. 2.1) and STX260 (R.F. 2.6), and not to 2-MeOE2 (STX66; R.F. 1.1) or STX641 (R.F. 0.9), in comparison to MCF-7wt cells.

To establish whether BCRP has an effect on the efficacy of STX140 *in vivo*, dual tumour xenograft studies were set up. Nude mice were inoculated on one flank with the parental cell line, and on the other with the BCRP-expressing derivative to ensure that a direct comparison was achieved. In the first instance, use of an A2780wt/A2780.140 model was explored. An initial study using the A2780wt and A2780.140 cells as dual xenografts was unsuccessful as the ovarian cells had a variable take rate, and those of the control group grew very rapidly, forming ulcerated tumours. Despite this, it could be seen that both STX140 and MXR were highly efficacious in the A2780wt tumours (Supplementary Data A), whereas in the A2780.140 tumours, there was an indication that the efficacy of MXR may be lower than that of STX140 (Supplementary Data B). RT–PCR analysis of mRNA from tumours taken at the end of study indicated that none of the treatments affected the amount of BCRP expressed by either tumour type (Supplementary Data C).

The dual tumour model was repeated using the MCF-7wt and MCF-7.MR cells (Figure 5). Both the MCF-7wt and MCF-7.MR control tumours grew at similar rates over the 35 day treatment period, to around 400% of their starting volume. Treatment with STX140 at 20 mg kg^−1^/day completely inhibited the growth of the MCF-7wt tumours (*P*<0.001), and treatment with either 1 or 2.5 mg kg^−1^ MXR twice weekly also significantly inhibited the growth of the MCF-7wt tumours (*P*<0.05), although to only ∼50% of the volume of the control tumours (Figure 5A). In contrast, neither dose of MXR had any effect on tumour growth in the BCRP-expressing tumours (Figure 5B), whereas STX140 again completely inhibited growth (*P*<0.001), and by day 35 resulted in tumour regression to 78% of the original starting volume. No significant weight loss was seen in any of the animals over the duration of the study (data not shown).

To ascertain whether either transfer of the cells to the *in vivo* setting or treatment with the compounds had affected the expression of BCRP in either the MCF-7wt or MCF-7.MR cells, tumours were removed at the end of the study for RT–PCR analysis (Figure 5C). In all cases, expression remained low in the MCF-7wt tumours and was comparable to that in the MCF-7wt cells cultured *in vitro*, whereas expression of BCRP in the MCF-7.MR tumours was several-fold higher. Although BCRP mRNA expression in the MCF-7.MR tumours *in vivo* is apparently lower than that in MCF-7.MR cells *in vitro*, this decrease may be due to the infiltration of other cell types, such as those forming visible blood vessels, into the MCF-7.MR tumour over the duration the study.

## Discussion

STX140 and other 2-substituted oestrogen sulphamates are highly effective at inhibiting the growth of cancer cells and angiogenesis, both *in vitro* and *in vivo* (Chander *et al*, 2007; Newman *et al*, 2007; Foster *et al*, 2008). Previous work has indicated that these compounds are also active against cell lines and tumours resistant to other chemotherapeutic regimens, including those which express P-glycoprotein (Day *et al*, 2003; Suzuki *et al*, 2003; Newman *et al*, 2008). To investigate possible mechanisms of resistance to the 2-substituted oestrogen sulphamates, and to elucidate further the mechanism of action of these compounds, long-term dose escalation was used to derive a sub-line of A2780 ovarian cancer cells, A2780.140, resistant to STX140.

A2780 cells are frequently used in resistance studies. Their susceptibility to resistance development has been attributed to a 0.01% subpopulation of mismatch repair defective A2780 cells (McLaughlin *et al*, 1991; Aquilina *et al*, 2000). A2780 sub-lines have been derived with resistance to many chemotherapeutic compounds including cisplatin (Behrens *et al*, 1987), taxol (Ferlini *et al*, 2003), both cisplatin and taxol (Pratesi *et al*, 2003) and DOX (Hamilton *et al*, 1984). STX140-resistant sub-lines could not be derived from MCF-7, PC-3 and LNCaP cells, all which have similar sensitivities to STX140 as A2780 cells.

Immunoblot and RT–PCR analyses demonstrated that BCRP expression is dramatically increased in the A2780.140 cells. BCRP is a transmembrane ABC transporter protein expressed, apparently as a protective mechanism, in many healthy tissues including the placenta, testes, gut and blood brain barrier (Maliepaard *et al*, 2001; Dietrich *et al*, 2003), as well as in stem cells (Zhou *et al*, 2001, 2002). It is able to transport porphyrins, such as haem, and is upregulated in hypoxic conditions, suggesting that it is essential for the survival of stem cells under low-oxygen conditions (Krishnamurthy and Schuetz, 2006). In healthy breast tissue it appears to concentrate both vitamins and xenobiotics into breast milk (van Herwaarden *et al*, 2007).

The high level of BCRP expression in the A2780.140 sub-line appears to be relatively stable, as it is sustained when the cells are cultured in the absence of STX140 for over eight weeks. Its activity was demonstrated by the use of substrate accumulation FACS analysis, using MXR, a BCRP substrate, in the presence and absence of a BCRP inhibitor, novobiocin. However, the resistance of the A2780.140 cells to STX140 is low, only increasing by eight times compared to the A2780wt cells, and they remain sensitive to STX140 at higher doses. This compares with resistance factors of >100 for many cell lines resistant to other compounds (for example, A2780 cells resistant to gemcitabine have an R.F. of 150 000; Ruiz van Haperen *et al*, 1994). The cells are partially resistant to DOX, as reported for the MCF-7.MR cell line which also over-expresses BCRP (Taylor *et al*, 1991).

The substrate profile of BCRP, encompassing chemotherapeutic agents, xenobiotics and steroids, is continuing to expand and has been confused by the discovery of several variants of BCRP. A mutation at amino acid 482 is known to alter the specificity of BCRP for its substrates (Honjo *et al*, 2001). In wild-type BCRP, derived from normal tissues, an arginine is present at 482, and this form can also be found in cancer cell lines selected for resistance to chemotherapeutic agents. However, R482T and R482G mutations are found in the BCRP expressed by two well-characterised resistant cancer cell lines, MCF-7/AdrVp3000 (Lee *et al*, 1997) and S1-M1-80 (Miyake *et al*, 1999), respectively, selected by DOX and MXR treatment. Several polymorphisms in the ABCG2 gene may have further impact on BCRP substrate specificity, cellular localisation, and expression levels (Lemos *et al*, 2008). Sequencing confirmed that the form expressed by A2780.140 cells carries the wild-type arginine residue at position 482.

Of a large panel of other 2-substituted oestrogen sulphamates, the A2780.140 sub-line is cross-resistant only to the two most similar compounds. These compounds, STX243 and STX260, differ from STX140 in their 2-position substituents. Use of a well-characterised BCRP-expressing cell line, MCF-7.MR, further confirmed that the resistance of the A2780.140 cell line is BCRP-mediated, as the two cell lines have the same profile of sensitivity to MXR and the 2-substituted oestrogen sulphamates.

Immunoblot and RT-PCR analyses indicated that A2780.140 and MCF-7.MR cell lines have similar high expression of BCRP, despite MCF-7wt cells expressing low but measureable levels of BCRP, as previously noted (Robey *et al*, 2007), and A2780wt cells having no detectable BCRP expression. BCRP is a glycosylated protein, and the appearance of the BCRP immunoblot bands from the two cell lines is slightly different, suggesting a change in the glycosylation of the BCRP from the two sources. However, as the authors have demonstrated in these studies, this possible difference in glycosylation does not appear to affect the function of this protein. It has previously been shown that altered glycosylation of BCRP does not affect its localisation at the plasma membrane (Mohrmann *et al*, 2005).

As the expression of BCRP only alters the sensitivity of the cells to the 2-substituted oestrogen sulphamates which have a sulphamate group at the 17-position of the steroid D-ring, this suggests that the presence of this group is necessary for recognition of this class of compounds by BCRP. However, the sulphamates studied in this paper are derivatives of oestradiol, and many steroids, both unconjugated and conjugated to sulphate or glucuronide, including oestrogens, phytoestrogens and androgens, are substrates of BCRP (Imai *et al*, 2003; Velamakanni *et al*, 2007). Studies have also shown that steroids and cholesterol, and steroid agonists and antagonists such as tamoxifen and diethylstilboestrol, can modulate the expression of BCRP without themselves being substrates (Imai *et al*, 2002; Janvilisri *et al*, 2003; Sugimoto *et al*, 2003; Morita *et al*, 2005; Pavek *et al*, 2005). A novel oestrogen response element (ERE) has been found in the human BCRP promoter (Ee *et al*, 2004a, 2004b), although it has also been suggested that oestradiol post-transcriptionally downregulates BCRP (Imai *et al*, 2005). Despite these observations, in this study the increased expression of BCRP in A2780.140 cells after long-term exposure to STX140 cannot be mediated by the ER as the A2780 cell line is ER negative and STX140 is non-oestrogenic (Chander *et al*, 2007).

To study the effect that the expression of BCRP has on the efficacy of STX140 *in vivo*, we used a dual xenograft tumour mouse model similar to that established in our laboratory to study the effect of P-glycoprotein expression on STX140 efficacy (Newman *et al*, 2008). Wild-type and resistant cells were inoculated in Matrigel on opposite flanks, allowing direct comparison of the treatment effects on the two xenografts whilst reducing the number of animals required for each study. An initial study using the A2780wt and A2780.140 cells as dual xenografts was unsuccessful as the ovarian cells had a variable take rate, and grew very rapidly forming ulcerated tumours. However, in a repeat study using an MCF-7wt/MCF-7.MR dual xenograft model, both tumour types grew steadily at similar rates. Despite the BCRP-expressing cells having a similar level of resistance to STX140 and MXR *in vitro*, STX140 was highly active *in vivo* in both MCF-7wt and MCF-7.MR tumours, significantly inhibiting their growth, whereas MXR, a chemotherapeutic agent in clinical use, inhibited the growth of the wild-type xenografts, but was inactive in those which expressed BCRP.

It has previously been shown that the plasma concentration of STX140, when dosed orally to mice at 10 mg kg^−1^, is maintained at 1 *μ*M or above for 24 h due to its excellent oral bioavailability, with peak concentration at ∼8.5 *μ*M 3 h after dosing (Ireson *et al*, 2004). In this study the mice were given 20 mg kg^−1^ per day STX140, suggesting that the concentration of STX140 would be maintained at above 1 *μ*M in the plasma. As the IC_50_ of STX140 in BCRP-expressing cells *in vitro* is only 1.5–2 *μ*M, this dose may be regularly or continuously exceeded in the *in vivo* model at a 20 mg kg^−1^ per day dose, resulting in good efficacy of STX140 in BCRP-expressing tumours *in vivo*. In addition to its anti-proliferative properties, the anti-angiogenic qualities of STX140 also contribute to its efficacy *in vivo*, as STX140 has been shown to cause significant inhibition of angiogenesis when dosed at 10 mg kg^−1^ in a Matrigel plug assay (Chander *et al*, 2007) and at 20 mg kg^−1^ in xenograft models (Chander *et al*, 2007; Foster *et al*, 2008). This property would be unaffected by BCRP expression in the cancer cells of the tumour, adding to the efficacy of STX140 in BCRP-expressing tumours.

The sensitivity of the BCRP-expressing tumours to the effects of STX140 indicates that STX140 would be of therapeutic benefit in cancers observed to have BCRP-mediated resistance to chemotherapy. BCRP expression has been seen in around 40% of human tumours pre-treatment, and is most prevalent in tumours of the digestive tract and in haematological malignancies. In adult lymphoblastic leukaemia BCRP expression is associated with shorter disease-free survival, and in acute myeloid leukaemia several studies have indicated that there is a positive correlation between its expression and resistance (Krishnamurthy and Schuetz, 2006).

In conclusion, despite long-term dose escalation of STX140 in A2780 ovarian cancer cells resulting in a sub-line which expresses high levels of wild-type BCRP, STX140, in contrast to MXR, is highly efficacious *in vivo* in both wild-type and BCRP-expressing tumour xenografts. STX140 has also previously been shown to be efficacious against tumours which express P-glycoprotein, and against drug-resistant patient breast cancer xenografts which do not express P-glycoprotein. The combination of these properties with its excellent oral bioavailability and lack of toxicity indicate that STX140, currently in pre-clinical development, is a promising therapeutic agent for both non-drug-resistant cancers, and for cancers which have developed resistance to other agents.

## Figures and Tables

**Table 1:** The effect of various compounds on the proliferation of A2780wt and A2780.140 cells in the presence and absence of novobiocin, a BCRP inhibitor

	**IC_50_ (nM)**			
**Compound**	**A2780wt**	**A2780.140**	**R.F. (no BCRP inhibitor)**	**R.F. (+60 *μ*M novobiocin)**
STX66	500	590	1.2	nd
STX68	410	380	0.9	nd
STX138	400	400	1.0	nd
STX140	240	1930	7.9	1.5
STX243	260	830	3.2	nd
STX260	230	1170	5.0	1.9
STX640	320	320	1.0	nd
STX641	40	50	1.2	1.2
STX564	180	170	0.9	nd
Taxol	6	4	0.7	nd
Colchicine	4	5	1.1	nd
MXR	150	940	6.5	2.4
DOX	170	570	3.3	1.2

Cells were treated in triplicate with a range of concentrations of the compounds in the presence or absence of 60 μM novobiocin. After 4 days proliferation was measured using the CellTiter96 Aqueous One assay (Promega) and the IC_50_ values were calculated. Results are representative of at least two separate experiments (IC_50_=nM; R.F.=resistance factor (=IC_50_ A2780.140/IC_50_ A2780wt); nd=not determined).

**Table 2:** The effect of various compounds on the proliferation of MCF-7wt and MCF-7.MR cells

	**IC_50_ (nM)**		
**Compound**	**MCF-7wt**	**MCF-7.MR**	**R.F.**
STX66	2980	3360	1.1
STX68	230	280	1.2
STX140	240	1530	6.4
STX243	210	440	2.1
STX260	300	780	2.6
STX640	200	160	0.8
STX641	70	60	0.9
MXR	180	970	5.4

Cells were treated in triplicate with a range of concentrations of the compounds. After 4 days proliferation was measured using the CellTiter96 Aqueous One assay (Promega) and the IC_50_ values were calculated. Results are representative of at least two separate experiments (IC_50_=nM; R.F.=resistance factor (=IC_50_ MCF-7.MR/IC_50_ MCF-7wt)).
