# Supplementary figures and images for: BCRP expression does not result in resistance to STX140 in vivo, despite the increased expression of BCRP in A2780 cells in vitro after long-term STX140 exposure
Source: Br J Cancer. 2009 Jan 20;100(3):476–86. doi: 10.1038/sj.bjc.6604873 (PMC2658539; doi:10.1038/sj.bjc.6604873)

## Slide 1
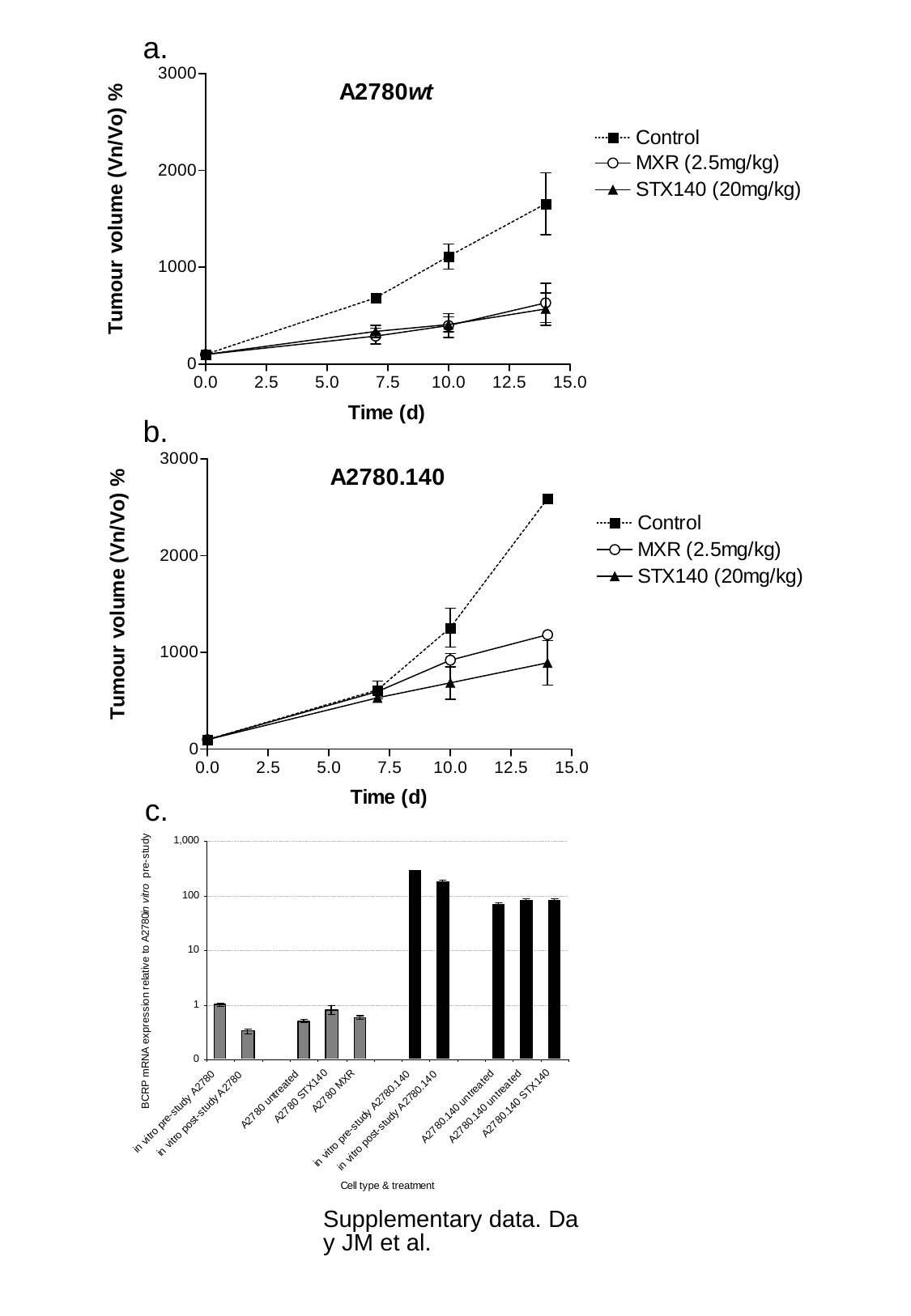

a.
b.
c.
Supplementary data. Day JM et al.

Supplement: Supplementary Data [file 6604873x1.ppt]
