# Supplementary material for: BCRP expression does not result in resistance to STX140 in vivo, despite the increased expression of BCRP in A2780 cells in vitro after long-term STX140 exposure
Source: Br J Cancer. 2009 Jan 20;100(3):476–86. doi: 10.1038/sj.bjc.6604873 (PMC2658539; doi:10.1038/sj.bjc.6604873)
Supplement: Supplementary Data Legend [file 6604873x2.doc]

**Supplementary data**

**Dual tumour A2780wt & A2780.140 xenograft model.**

Female MF-1 nu/nu mice were injected s.c. in one flank with 5 x 10^6^ A2780wt cells and in the other with 5 x 10^6^ A2780.140 cells in ice-cold Matrigel (n = 6 per group). Daily oral administration of STX140 vehicle (0.1ml 10% THF / 90% propylene glycol), STX140 (20mg/kg), or twice weekly i.v. administration of MXR (2.5mg/kg in saline) was initiated when the tumours reached 50 to 150mm^3^ in volume (Day 0).

*a.* A2780wt tumour growth: Dosing with either STX140 or MXR resulted in inhibition of tumour growth compared to control.

*b*. A2780.140 tumour growth: Dosing with either STX140 or MXR resulted in inhibition of tumour growth compared to control, however, in this cell line STX140 tended towards higher efficacy than MXR.

*c*. BCRP mRNA expression: RT-PCR analysis, using Taqman expression assays for BCRP, and for an endogenous control gene, RPLO, of mRNA extracted from A2780wt and A2780.140 tumours at the end of the study.
